# Reference values for lipid profile in Iranian children and adolescents: the CASPIAN-V study

**DOI:** 10.1186/s12944-020-1186-1

**Published:** 2020-01-31

**Authors:** Fatemeh Azizi-Soleiman, Maliheh Khoramdad, Ramin Heshmat, Hanieh-Sadat Ejtahed, Mohammad Esmaeil Motlagh, Seyede Shahrbanoo Daniali, Mostafa Qorbani, Roya Kelishadi

**Affiliations:** 1 School of Health, Arak University of Medical Sciences, Arak, Iran; 2 Department of Epidemiology, School of Public Healthz, Iran University of Medical Sciences, Tehran, Iran; 3 Chronic Diseases Research Center, Endocrinology and Metabolism Population Sciences Institute, Tehran University of Medical Sciences, Tehran, Iran; 4 Obesity and Eating Habits Research Center, Endocrinology and Metabolism Clinical Sciences Institute, Tehran University of Medical Sciences, Tehran, Iran; 5 Department of Pediatrics, Ahvaz Jundishapur, University of Medical Sciences, Ahvaz, Iran; 6 Department of Pediatrics, Child Growth and Development Research Center, Research Institute for Primordial Prevention of Non-Communicable Disease, Isfahan University of Medical Sciences, Isfahan, Iran; 7 Non-communicable Diseases Research Center, Alborz University of Medical Sciences, Karaj, 31485/56 Iran; 8 Endocrinology and Metabolism Research Center, Endocrinology and Metabolism Clinical Sciences Institute, Tehran University of Medical Sciences, Tehran, Iran

**Keywords:** Lipid profile, Pediatrics, Reference value

## Abstract

**Background:**

We aimed to develop the age- and sex-specific reference values for lipid profile of Iranian pediatric population.

**Methods:**

Fasting lipid profiles of 3843 participants, aged 7 to 18 years, were extracted from a surveillance survey on Iranian children and adolescents living in 30 provinces across the country.

**Results:**

The mean (SD) age of participants was 12.3(3.1) years, and 52.3% of them were boys. Significant differences were observed between genders comparing the levels of triglyceride (TG) (*P* = 0.04), total cholesterol (TC) (*P* = 0.02), low-density lipoprotein- cholesterol (LDL-C) (*P* = 0.01), and non-high-density lipoprotein cholesterol (non-HDL-C) (*P* = 0.03). In both genders, TG levels increased with age in the 75th and higher percentiles. Among boys, TC showed a decreasing trend at all percentiles and all age groups. In girls, TC levels increased with age at all percentiles except for the 75th and 90th percentiles. Among boys, the levels of LDL-C and HDL-C decreased with age in all percentiles. However, LDL-C and HDL-C concentrations increased up to the 50th percentile in girls and then decreased with age. The non-HDL-C level decreased in the 50th and higher percentiles among boys and in the 90th and 95th percentiles among girls. The TG/HDL-C ratio increased with age at all percentiles in boys. In girls, TG/HDL-C ratio increased with age in the 50th and higher percentiles.

**Conclusions:**

Based on the observed differences, it seems necessary to determine age- and sex-specific cut-off values for lipid parameters of children and adolescents in different populations.

## Background

Dyslipidemia is one of the common problems in children and adolescents. This condition is defined as disorders of triglyceride (TG), high-density lipoprotein cholesterol (HDL-C), and low-density lipoprotein cholesterol (LDL-C). The non-HDL-C, which encompasses all atherogenic cholesterols suggested as a novel predictor of cardiovascular diseases (CVDs) which has been linked to some indicators of metabolic syndrome in adolescents [1].

Dyslipidemia can be developed in childhood and might lead to atherosclerotic lesions in adulthood [2] such as increased intima-media thickness [3]. Genetic form of hypercholesterolemia or familial hypercholesterolemia (FH) is also an important issue in children as it can increase risk of CVDs later in life [4]. This disorder has two forms characterized by very high levels of LDL-C. Heterozygous form of FH is more prevalent and results in atherosclerotic signs like increased carotid intima-media thickness from childhood [5]. Homozygous FH is also related to premature atherosclerotic CVDs and early death in children [6]. Although homozygous form of FH is rare, but it is associated with extremely high risk of CVDs. Its early detection and treatment can reduce the risk of cardiovascular events. Hence, the diagnosis of dyslipidemia is an important health issue in children and adolescents.

Lipid levels change during different periods of childhood and are affected by gender and ethnicity; therefore, lipid abnormalities in the pediatric age group should be identified based on the percentile thresholds. According to the guidelines of the National Cholesterol Education Program (NCEP) and the Bogalusa Heart Study, TC, LDL-C, non-HDL-C, and TG values of >75th percentile are considered as borderline. Furthermore, values of >95th percentile for the aforementioned parameters and < 10th percentile for HDL-C are considered as abnormal [3, 7]. It was previously recommended to screen dyslipidemia in children ≥2 years old who have a first or second -degree relative (males < 55 and females < 65 years old) with confirmed CVDs or those with established risk factors of CVDs such as overweight, diabetes, hypertension, smoking, unhealthy diet, and inactivity [8]. According to the American Heart Association Statement published in 2019, identification and treatment of CVD in youth should be initiated by defining the risk level [9]. This process is based on the existence of the following factors: children with traditional CVD risk factors including FH, as well as childhood cancer, endocrine and renal diseases, inflammatory disorders, underlying heart disease, and non-communicable diseases. Thus, the risk assessment should be done by evaluating the lipid and glucose profile, smoking status, family history of premature CVD, as well as levels of BP, BMI, and physical activity.

A systematic review in 2015 showed relatively high prevalence of dyslipidemia among Iranian children and adolescents [10]. Hence, it is of particular importance for healthcare professionals to identify children at risk of atherosclerosis and release of guidelines for controlling and preventing CVDs in the future.

Studies in different populations and ethnic groups have revealed variable cholesterol concentrations [11]. It is reported that whites have higher levels of very low density lipoprotein cholesterol (VLDL-C) and TG, as well as, slightly lower HDL-C levels compared with blacks [12]. The differences in factors like body fat distribution, activity of enzymes involved in lipid hydrolysis, insulin response, and some specific apolipoproteins can partly explain the interethnic differences in serum lipids [13]. Many countries use the NCEP pediatric cutpoints for screening the lipid disorders without consideration of possible ethnic differences. Therefore, the diagnostic criteria of dyslipidemia and reference values of lipid parameters should be refined based on the nationality, ethnicity, as well as the age and sex of individuals. This is especially important among children and adolescents. In the present study, we aimed to develop the age- and sex-specific reference values for serum lipid parameters in a representative population of Iranian children and adolescents.

## Methods

### Study population

The Ethics Committee of the Isfahan University of Medical Sciences approved this study. Verbal and written informed consents were obtained from all participants and their parents, respectively. The data was extracted from the fifth survey of a surveillance program entitled Childhood and Adolescence Surveillance and Prevention of Adult Non communicable disease (CASPIAN-V). It was conducted as a nationwide study on a representative sample population of urban and rural Iranian children with 7–18 years of age, who lived in 30 provinces across the country. Details of this study is published elsewhere [14].

Lipid profiles (TG, TC, LDL-C, HDL-C, and non-HDL-C) of 3843 children were obtained. Children and adolescents with history of any underlying health problem or chronic medication use were not included in the study.

### Measurements

Venous blood samples were obtained from all the subjects between 7 and 9 A.M and following overnight fasting. The collected samples were centrifuged and frozen at − 70 °C. Serum lipid parameters including TC, LDL-C, HDL-C, and TG were measured by an enzymatic method colorimetric method using an automated analyzer (Hitachi Automatic Analyzer 7600, Hitachi). LDL-C was measured directly. Firstly, we used cholesterol esterase and cholesterol oxidase to produce H2O2, which was consumed by catalase in the presence of 4-aminoantipyrene. Then, N, N-bis-(4-sulfobutyl)-mtoluidine disodium salt, buffer, and a detergent were added to release cholesterol from LDL-C and a colored product that is proportional to LDL-C concentration. Non-HDL-C was calculated as “TC- HDL-C”.

### Statistical analysis

Continuous and categorical variables were expressed as mean (standard deviation, SD) and number (%), respectively. The mean values of continuous variables were compared between genders using independent samples Student t test. A parametric method was used to define the age-specific reference values by obtaining smooth centile curves and explicit formulae for the centile estimates and SD scores (age-sex standardized values). Each parameter of exponential-normal or modulus-exponential-normal density was modeled as a fractional polynomial function of age. The estimation was based on the maximum likelihood. These three- and four-parameter models transformed the data to attain normal distribution and remove skewness and/or kurtosis. Fractional polynomials provide more flexible curves than those of conventional polynomials. Goodness of fit was assessed using Q-Q plots and Shapiro-Wilk W-test of the SD scores. Likelihood ratio test was applied as an enlarged model.

## Results

**Fig. 1:**
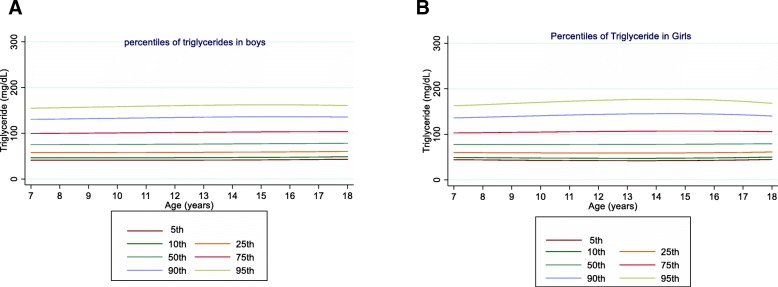
Reference percentile curves for TG according to age in Iranian boys (**a**) and girls (**b**)

The mean (SD) age of participants was 12.3 (3.1) years, and 52.3% of them were boys. Table 1 shows the mean, SD, and percentile values for lipid parameters in the studied population according to their age and gender. The mean value of TG was 85.46 (39.19) mg/dL in boys and 88.04 (41.31) mg/dL in girls (*P* = 0.04). In both genders, TG levels increased with age in the 75th and higher percentiles. Furthermore, girls had higher TG levels than boys in all age groups (Fig. 1).

**Table 1:** Reference values for lipid profile according to sex and age groups: the CASPIAN-V study

Age	n	Mean	SD	Percentile							Age	n	Mean	SD	Percentile						
				5	10	25	50	75	90	95					5	10	25	50	75	90	95
Boys											Girls										
Triglyceride (mg/dL)											Triglyceride (mg/dL)										
7	64	78.59	33.68	41.11	46.44	57.64	74.86	99.34	130.33	154.77	7	76	85.07	37.89	44.04	49.08	59.87	77.26	102.90	135.91	162.59
8	135	84.16	41.71	41.06	46.42	57.69	75.05	99.76	131.11	155.85	8	159	88.01	38.27	43.57	48.67	59.59	77.29	103.53	137.49	165.06
9	158	84.89	41.21	41.02	46.42	57.76	75.27	100.23	131.94	157.01	9	187	87.94	44.62	43.11	48.25	59.33	77.34	104.19	139.14	167.65
10	204	85.85	37.40	41.01	46.44	57.87	75.51	100.72	132.80	158.19	10	158	84.31	35.7	42.68	47.87	59.08	77.40	104.86	140.80	170.24
11	201	89.63	42.03	41.04	46.50	58.00	75.79	101.23	133.66	159.35	11	176	88.65	42.62	42.31	47.55	58.89	77.49	105.51	142.35	172.65
12	213	82.37	36.30	41.11	46.59	58.17	76.08	101.73	134.46	160.41	12	228	87.28	42.71	42.04	47.32	58.76	77.61	106.09	143.70	174.72
13	257	83.22	37.43	41.23	46.74	58.38	76.40	102.22	135.17	161.31	13	230	91.22	44.03	41.90	47.21	58.74	77.75	106.56	144.71	176.24
14	173	85.16	39.61	41.42	46.95	58.64	76.73	102.65	135.73	161.97	14	173	88.94	44.04	41.94	47.26	58.83	77.93	106.89	145.26	177.00
15	132	87.88	41.75	41.68	47.24	58.95	77.08	103.02	136.09	162.31	15	84	90.90	43.25	42.18	47.51	59.08	78.15	107.02	145.22	176.77
16	208	87.37	39.28	42.05	47.61	59.33	77.43	103.29	136.21	162.25	16	134	89.27	39.34	42.69	48.01	59.51	78.41	106.91	144.44	175.34
17	170	87.77	41.92	42.52	48.08	59.77	77.79	103.45	136.01	161.71	17	133	83.60	37.61	43.52	48.79	60.16	78.72	106.49	142.80	172.50
18	87	85.08	33.93	43.11	48.66	60.29	78.14	103.45	135.44	160.61	18	87	90.39	40.29	44.74	49.94	61.07	79.08	105.74	140.18	168.10
total	2002	85.46	39.19	41.46	46.95	58.51	76.36	101.86	134.32	160.01	total	1825	88.04	41.31	42.71	47.95	59.26	77.79	105.63	142.15	172.13
Total cholesterol (mg/dL)											Total cholesterol (mg/dL)										
7	65	157.0	29.77	106.42	117.18	135.16	154.33	175.04	197.75	213.35	7	77	155.87	30.26	105.00	115.13	132.49	151.88	172.77	194.41	194.41
8	136	153.35	29.48	109.19	119.37	136.22	153.99	173.02	193.68	207.69	8	157	152.19	25.32	108.15	117.85	134.32	152.55	172.01	192.01	205.08
9	159	154.18	29.60	110.66	120.48	136.64	153.59	171.63	191.12	204.28	9	184	157.78	28.38	110.60	119.95	135.72	153.05	171.43	190.20	202.40
10	202	155.28	24.18	111.27	120.88	136.64	153.13	170.61	189.46	202.14	10	158	152.84	26.73	112.44	121.52	136.76	153.43	171.01	188.88	200.46
11	203	155.61	28.50	111.30	120.79	136.34	152.59	169.81	188.33	200.79	11	177	153.58	23.71	113.75	122.63	137.50	153.71	170.74	188.00	199.15
12	212	153.20	26.03	110.91	120.36	135.83	152.00	169.12	187.54	199.93	12	228	156.55	4.26	114.59	123.35	137.99	153.91	170.61	187.50	198.39
13	254	155.64	24.30	110.24	119.63	135.16	151.33	168.48	186.94	199.36	13	230	156.25	25.28	115.01	123.72	138.26	154.06	170.62	187.34	198.12
14	170	151.06	24.31	109.36	118.82	134.36	150.61	167.86	186.45	198.96	14	173	151.34	25.47	115.06	123.78	138.34	154.16	170.74	187.48	198.28
15	134	154.91	26.54	108.33	117.83	113.46	149.83	167.23	186.01	198.67	15	84	155.47	22.96	114.78	123.56	138.25	154.22	170.98	187.91	198.84
16	210	146.83	27.72	107.19	116.74	132.48	149.00	166.58	185.59	198.42	16	134	155.26	25.88	114.21	123.11	138.02	154.25	171.32	188.60	199.77
17	169	146.65	25.36	105.97	115.58	131.43	148.11	165.89	185.15	198.17	17	134	158.00	24.22	113.39	122.44	137.65	154.26	171.76	189.53	201.03
18	88	156.22	29.13	104.70	114.36	130.34	147.17	165.16	184.69	197.90	18	88	153.32	30.46	112.33	121.58	137.16	154.24	172.29	190.68	202.61
total	2002	153.02	26.87	109.23	118.86	134.70	151.31	168.97	188.04	200.91	total	1824	154.93	25.87	112.84	121.88	137.08	153.68	171.20	189.00	189.00
Low-density lipoprotein cholesterol (mg/dL)											Low-density lipoprotein cholesterol (mg/dL)										
7	66	93.95	27.44	54.72	62.06	74.74	88.90	104.49	121.42	132.92	7	77	92.39	23.49	51.70	59.28	72.78	88.67	106.18	124.13	135.87
8	136	88.08	22.98	55.17	62.43	74.93	88.84	104.11	120.65	131.85	8	159	88.65	22.57	54.27	61.51	74.22	88.94	104.89	121.02	131.45
9	159	89.90	23.96	55.61	62.78	75.09	88.75	103.70	119.84	130.75	9	187	91.69	24.39	55.85	62.89	75.15	89.22	104.35	119.49	129.24
10	202	90.34	19.17	55.99	63.07	75.21	88.63	103.27	119.04	129.68	10	159	89.14	24.61	56.82	63.76	75.78	89.50	104.17	118.82	128.21
11	202	90.16	21.60	56.27	63.28	75.25	88.45	102.83	118.28	128.69	11	177	89.97	19.55	57.42	64.31	76.22	89.77	104.22	118.63	127.84
12	212	89.06	20.56	56.40	63.34	75.19	88.23	102.41	117.62	127.85	12	228	92.81	19.66	57.77	64.65	76.52	90.02	104.39	118.70	127.85
13	257	91.36	20.66	56.33	63.23	75.00	87.95	102.01	117.09	127.22	13	230	91.91	20.94	57.94	64.83	76.71	90.21	104.59	118.90	128.05
14	173	87.32	21.91	56.01	62.90	74.65	87.59	101.66	116.74	126.88	14	173	89.69	19.55	57.99	64.89	76.81	90.35	104.78	119.14	128.32
15	134	90.78	22.94	55.40	62.31	74.13	87.17	101.37	116.63	126.91	15	84	90.25	19.76	57.92	64.84	76.80	90.41	104.90	119.34	128.58
16	210	85.34	21.87	54.44	61.41	73.39	86.66	101.17	116.82	127.39	16	134	90.82	21.53	57.75	64.69	76.69	90.36	104.93	119.45	128.75
17	169	84.75	20.25	53.09	60.18	72.41	86.06	101.07	117.36	128.42	17	134	92.89	20.71	57.48	64.44	76.47	90.18	104.83	119.43	128.79
18	88	94.43	24.39	51.34	58.58	71.17	85.36	101.10	118.33	130.09	18	88	89.18	25.71	57.11	64.07	76.12	89.87	104.56	119.23	128.63
total	2008	89.21	21.91	55.33	62.36	74.42	87.76	102.34	118.05	128.66	total	1830	90.88	21.71	56.89	63.87	75.98	89.82	104.63	119.44	128.95
High-density lipoprotein cholesterol (mg/dL)											High-density lipoprotein cholesterol (mg/dL)										
7	66	47.83	11.00	31.92	35.08	40.58	46.78	53.77	61.64	67.15	7	77	45.83	10.78	30.98	33.66	38.72	45.39	53.12	60.84	65.86
8	135	47.45	10.69	31.90	35.04	40.50	46.67	53.61	61.42	66.88	8	159	45.29	10.37	31.14	33.77	38.73	45.26	52.79	60.30	65.16
9	158	46.37	9.66	31.86	34.98	40.40	46.52	53.40	61.13	66.54	9	187	47.44	9.93	31.34	33.93	38.80	45.17	52.51	59.80	64.51
10	203	48.19	9.86	31.79	34.89	40.27	46.32	53.13	60.78	66.12	10	159	47.24	10.66	31.57	34.11	38.89	45.13	52.28	59.36	63.93
11	203	46.43	9.51	31.71	34.78	40.10	46.08	52.80	60.34	65.61	11	176	45.36	8.86	31.80	34.31	39.00	45.11	52.09	58.98	63.42
12	212	46.69	10.40	31.60	34.63	39.88	45.78	52.40	59.82	65.00	12	228	46.27	9.75	32.03	34.50	39.12	45.12	51.95	58.68	63.00
13	257	46.97	9.98	31.46	34.45	39.62	45.42	51.92	59.21	64.29	13	230	46.09	9.23	32.23	34.67	39.23	45.15	51.87	58.48	62.71
14	173	45.74	9.51	31.28	34.22	39.30	45.00	51.37	58.50	63.46	14	173	43.23	8.52	32.37	34.80	39.33	45.19	51.85	58.38	62.57
15	134	45.70	9.22	31.06	33.95	38.93	44.50	50.73	57.69	62.52	15	84	47.03	8.72	32.44	34.87	39.40	45.26	51.91	58.43	62.61
16	210	43.54	9.62	30.80	33.63	38.50	43.93	50.00	56.77	61.47	16	134	46.58	9.83	32.42	34.87	39.43	45.33	52.05	58.63	62.86
17	170	44.60	10.09	30.50	33.25	38.00	43.29	49.18	55.74	60.29	17	134	47.92	9.53	32.28	34.77	39.40	45.42	52.28	59.03	63.37
18	88	43.96	7.86	30.14	32.82	37.43	42.56	48.27	54.61	59.00	18	88	45.47	9.56	32.00	34.55	39.31	45.52	52.63	59.65	64.18
total	2009	46.14	9.89	31.36	34.34	39.49	45.27	51.75	59.01	64.07	total	1829	46.11	9.68	31.90	34.41	39.10	45.22	52.22	59.12	63.56
Non-high-density lipoprotein cholesterol (mg/dL)											Non-high-density lipoprotein cholesterol (mg/dL)										
7	66	111.33	32.02	62.13	71.54	87.87	106.14	126.40	148.71	164.00	7	76	110.84	24.27	67.31	75.78	90.26	106.32	123.70	142.02	154.20
8	136	105.47	27.01	66.29	75.00	89.79	105.94	123.45	142.33	155.06	8	157	106.94	23.03	66.12	77.33	91.27	106.61	123.06	140.27	151.62
9	159	107.45	26.62	68.45	76.75	90.69	105.73	121.84	139.03	150.53	9	183	110.67	25.15	70.24	78.32	91.97	106.90	122.85	139.47	150.42
10	203	107.85	22.44	69.40	77.49	91.00	105.49	120.94	137.34	148.26	10	159	106.32	26.15	70.97	78.97	92.47	107.20	122.89	139.19	149.92
11	202	108.61	25.79	69.62	77.63	90.97	105.25	120.44	136.53	147.24	11	177	108.02	21.44	71.44	79.41	92.84	107.47	123.04	139.20	149.81
12	211	105.73	22.93	69.43	77.42	90.74	105.00	120.17	136.24	146.94	12	228	110.27	22.17	71.73	79.70	93.11	107.71	123.23	139.34	149.91
13	256	108.05	22.51	68.99	77.01	90.40	104.74	120.04	136.26	147.06	13	230	110.15	23.06	71.89	79.86	93.28	107.89	123.42	139.52	150.09
14	167	103.68	21.60	68.41	76.49	90.00	104.50	120.00	136.46	147.45	14	173	107.91	23.71	71.94	79.92	93.36	107.99	123.55	139.68	150.27
15	134	109.20	26.03	67.77	75.92	89.57	104.28	120.02	136.79	148.00	15	84	108.44	21.44	71.88	79.88	93.34	108.00	123.58	139.76	150.73
16	210	103.28	24.96	67.12	75.33	89.15	104.08	120.10	137.20	148.66	16	132	107.31	20.39	71.72	79.72	93.20	107.88	123.49	139.70	150.35
17	169	102.10	23.09	66.74	74.7	88.76	103.91	120.22	137.68	149.40	17	132	111.09	22.69	71.45	79.45	92.92	107.61	123.24	139.47	150.14
18	88	112.26	27.30	65.85	74.23	88.40	103.79	120.39	138.22	150.20	18	88	107.85	27.19	71.06	79.04	92.50	107.17	122.80	139.03	149.70
total	2001	106.62	24.69	68.01	76.22	90.01	104.87	120.80	137.80	149.16	total	1819	108.89	23.34	71.07	79.10	92.65	107.45	123.22	139.62	150.40
Triglyceride to high-density lipoprotein cholesterol ratio											Triglyceride to high-density lipoprotein cholesterol ratio										
7	64	1.73	0.91	0.71	0.84	1.12	1.56	2.19	3.01	3.66	7	74	1.90	1.00	0.83	0.95	1.22	1.69	2.39	3.27	3.96
8	134	1.85	1.02	0.72	0.85	1.14	1.60	2.25	3.10	3.78	8	159	2.13	1.31	0.81	0.93	1.21	1.69	2.44	3.39	4.14
9	156	1.84	0.97	0.72	0.86	1.16	1.62	2.30	3.18	3.88	9	186	1.96	1.25	0.79	0.92	1.20	1.70	2.47	3.46	4.25
10	203	1.90	1.06	0.73	0.87	1.17	1.65	2.34	3.25	3.98	10	157	1.90	1.00	0.79	0.91	1.20	1.70	2.49	3.51	4.32
11	203	2.14	1.38	0.73	0.87	1.18	1.67	2.38	3.31	4.06	11	176	2.08	1.28	0.79	0.91	1.20	1.71	2.51	3.54	4.36
12	213	1.88	1.02	0.74	0.88	1.19	1.69	2.41	3.36	4.13	12	228	2.01	1.23	0.79	0.91	1.20	1.71	2.52	3.56	4.39
13	257	1.90	1.12	0.75	0.89	1.21	1.71	2.44	3.40	4.18	13	228	2.05	1.09	0.79	0.92	1.20	1.72	2.53	3.57	4.40
14	173	2.00	1.20	0.76	0.91	1.23	1.73	2.47	3.44	4.22	14	173	2.18	1.31	0.79	0.92	1.21	1.72	2.53	3.57	4.40
15	132	2.05	1.18	0.78	0.92	1.25	1.75	2.49	3.46	4.23	15	84	2.03	1.15	0.79	0.92	1.21	1.72	2.53	3.57	4.40
16	209	2.12	1.08	0.80	0.95	1.27	1.78	2.51	3.46	4.22	16	134	2.04	1.05	0.80	0.92	1.21	1.72	2.53	3.56	4.38
17	169	2.06	1.10	0.83	0.98	1.31	1.80	2.52	3.44	4.17	17	133	1.87	1.09	0.80	0.92	1.21	1.72	2.52	3.54	4.36
18	87	2.02	0.93	0.87	1.03	1.35	1.84	2.53	3.41	4.10	18	87	2.14	1.28	0.80	0.92	1.21	1.72	2.51	3.52	4.33
total	2000	1.97	1.11	0.76	0.90	1.21	1.70	2.41	3.34	4.08	total	1819	2.03	1.19	0.79	0.92	1.21	1.71	2.50	3.51	4.32

**Fig. 2:**
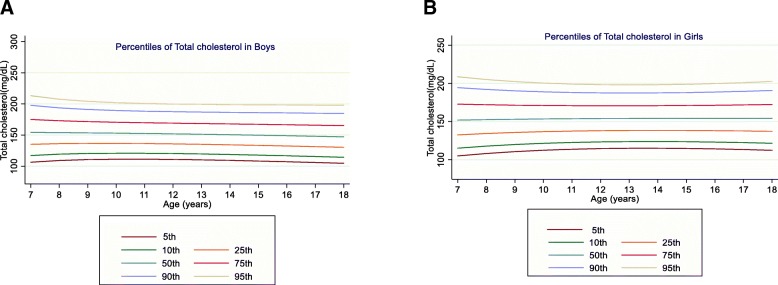
Reference percentile curves for TC according to age in Iranian boys (**a**) and girls (**b**)

The mean value of TC was 153.02 (26.87) mg/dL in boys and 154.93 (25.87) mg/dL in girls (*P* = 0.02). TC showed a decreasing trend at all percentiles and all age groups among boys. In girls, TC levels also increased with age at all percentiles except for the 75th and 90th percentiles (Fig. 2).

**Fig. 3:**
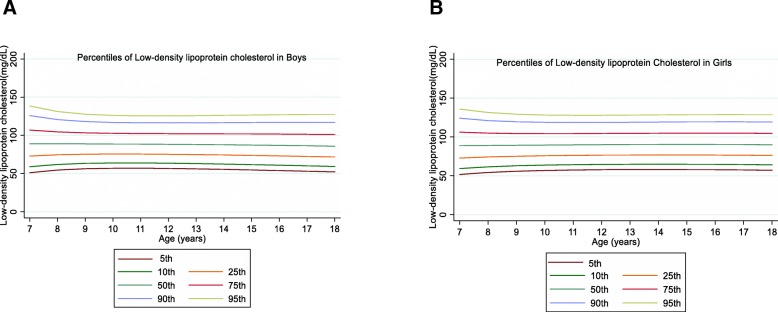
Reference percentile curves for LDL-C according to age in Iranian boys (**a**) and girls (**b**)

**Fig. 4:**
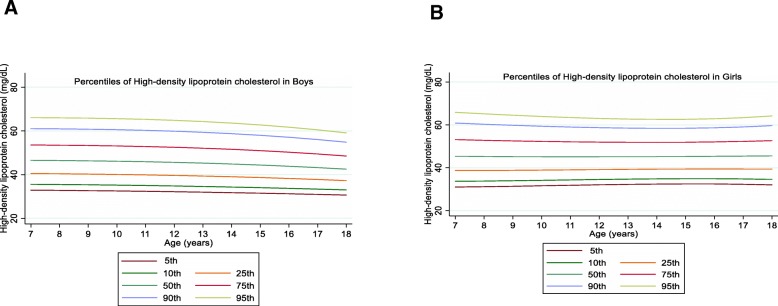
Reference percentile curves for HDL-C according to age in Iranian boys (**a**) and girls (**b**)

The overall mean value of LDL-C was 89.21 (21.91) mg/dL in boys and 90.88 (21.71) mg/dL in girls with a significant gender difference (*P* = 0.01). The overall mean value of HDL-C was 46.14 (9.89) mg/dL in boys and 46.11 (9.68) mg/dL in girls without significant gender difference (*P* = 0.92). Among boys, the levels of LDL-C and HDL-C decreased with age in all percentiles. However, LDL-C and HDL-C concentrations increased up to the 50th in girls and then decreased with age (Figs. 3 and 4).

**Fig. 5:**
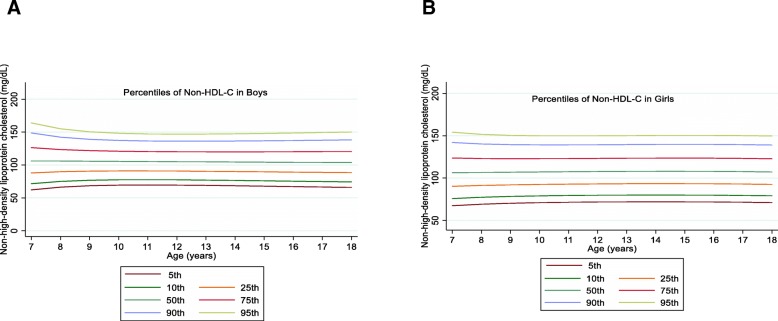
Reference percentile curves for non-HDL-C according to age in Iranian boys (**a**) and girls (**b**)

The overall mean value of non-HDL-C was 106.62 (24.69) mg/dL in boys and 108.89 (23.34) mg/dL in girls with a significant gender difference (*P* = 0.03). The non-HDL-C also decreased in the 50th and higher percentiles among boys and in the 90th and 95th percentiles among girls (Fig. 5).

**Fig. 6:**
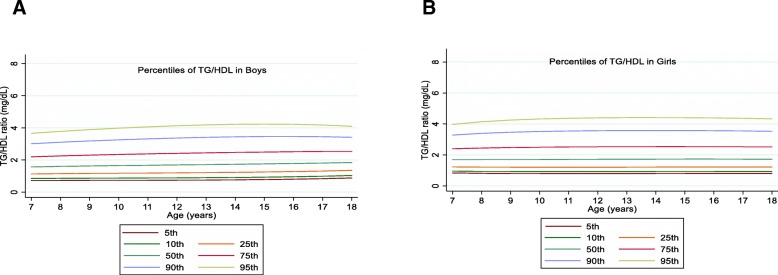
Reference percentile curves for the TG/non-HDL-C according to age in Iranian boys (**a**) and girls (**b**)

The overall mean value of the TG/HDL-C ratio was 1.97 (1.11) mg/dL in boys and 2.03 (1.19) mg/dL in girls without significant gender difference (*P* = 0.10). The TG/HDL-C ratio increased with age at all percentiles in boys. In girls, TG/HDL-C ratio increased with age in the 50th and higher percentiles (Fig. 6).

## Discussion

Our study provides reference values for serum lipid profiles in a nationally representative sample of Iranian pediatric population. In general, girls had higher levels of serum TG, TC, LDL-C, and non-HDL-C compared with boys. TG and TG/HDL-C ratio increased with age in both genders. As well, TC level increased with age in girls; whereas, HDL-C and non-HDL-C levels of both genders and TC in boys decreased with age.

Based on the reference values of lipid parameters released by the NCEP Expert Panel on Cholesterol Levels in Children, the following thresholds have been considered to define pediatric dyslipidemia: TC ≥ 200 mg/dL, LDL-C ≥ 130 mg/dL, HDL-C < 40 mg/dL, and non-HDL-C ≥ 145 mg/dL. Furthermore, the recommended thresholds for defining hypertriglyceridemia have been ≥100 mg/dL and ≥ 130 mg/dL in children aged 0–9 and 10–19 years, respectively [15]. In the present study, the 95th percentiles for TC and LDL-C concentrations were close to the values recommended by the NCEP. However, non-HDL-C level was slightly higher in present report. Furthermore, the 95th percentile of HDL-C was markedly lower, and that of TG was remarkably higher in the present study compared with the NCEP values. Variability in genetic background and different dietary habits can justify these differences.

Results of the current study demonstrated some gender differences in the reference values of serum lipid among children and adolescents. The pathophysiology, diagnosis and treatment of dyslipidemia in adults are different based on gender [16]. Moreover, several studies have reported gender differences in the levels of serum lipid parameters in children. A study on 1000 Indian students showed that boys had higher TC, LDL-C, and HDL-C and lower TG levels compared with girls [17]. A study on 2283 children and adolescents, aged 7–18 years, living in urban and rural areas of China revealed higher prevalence of elevated TC and LDL-C in girls than in boys. Furthermore, girls had higher mean values of HDL-C in comparison with boys [18]. Brazilian girls had also higher concentrations of TC and LDL-C compared with boys [19]. These variations may be in part due to the physiological and hormonal differences between boys and girls.

In line with other studies, we here found that some lipids parameters increased with age. In this regard, TG and TG/HDL-C in both genders and TC in girls increased with age in our study. In a study in Korean children, TG/HDL-C ratio increased with age in boys and decreased with age in girls [20]. In another study on 2141 Danish/North-European children and adolescents aged 6–19 years old, non-HDL-C levels decreased with age in boys [21]. In a longitudinal study on 633 children aged 8–18 years old, TC, LDL-C, and non-HDL-C levels in both genders and HDL-C in boys also increased with age. Moreover, TG level showed an increasing trend in boys during puberty [22].

Taking together, it is recommended to define the age and sex specific thresholds for evaluating lipid profile in the pediatric age group, especially among children and adolescents who are at risk of CVDs. In addition, it should be considered that factors such as ethnicity, nationality, dietary regimen, physical activity, hormonal status, environmental factors, and socioeconomic status can influence lipid profile in children [20, 23]. Therefore, establishing national references for lipid parameters should be encouraged.

This study has several strengths. To the best of our knowledge, this survey is one of the few efforts to establish reference values for lipid parameters in a large population-based sample of children and adolescents in the Eastern Mediterranean region; our sample was nationally- representative; and blood samples were drawn in fasting state. There are also some limitations. Our data was obtained based on a cross-sectional study; moreover we were not able to evaluate the impact of pubertal stage on lipid changes.

## Conclusion

This study showed different thresholds for lipid parameters in Iranian children and adolescents in comparison with those presented by the NCEP. We also observed some differences in the reference values between genders and among age groups. Since early detection of lipid disorders in children and adolescents using reference values from other nations and ethnicities may not be correctly performed, our results could provide nation-specific cutpoints for screening and interpretation of lipid abnormalities in Iranian pediatric population. However, further investigations should be conducted for defining optimal cutpoints to perform successful interventions and strategies to manage dyslipidemia in the pediatric age group.

## Data Availability

The datasets used and/or analyzed during the current study are available from the corresponding author on reasonable request.

## Abbreviations

CASPIAN: Childhood and Adolescence Surveillance and Prevention of Adult Noncommunicable disease
CVD: Cardiovascular diseases
HDL-C: High-density lipoprotein cholesterol
LDL-C: Low-density lipoprotein cholesterol
TG: Triglyceride
VLDL-C: Very low density lipoprotein cholesterol

## References

[1] Liu J, Wade T, Tan H (2007). Cardiovascular risk factors and anthropometric measurements of adolescent body composition: a cross-sectional analysis of the third National Health and nutrition examination survey. Int J Obes.

[2] Strand MF, Fredriksen PM, Hjelle OP, Lindberg M (2018). Reference intervals for serum lipids and prevalence of dyslipidaemia in 6–12-year-old children: The Health Oriented Pedagogical Project (HOPP). Scand J Public Health.

[3] Webber LS, Srinivasan SR, Wattigney WA, Berenson GS (1991). Tracking of serum lipids and lipoproteins from childhood to adulthood: the Bogalusa heart study. Am J Epidemiol.

[4] Marks D, Thorogood M, Neil HAW, Humphries SE (2003). A review on the diagnosis, natural history, and treatment of familial hypercholesterolaemia. Atherosclerosis.

[5] Braamskamp MJ, Langslet G, McCrindle BW, Cassiman D, Francis GA, Gagne C (2017). Effect of rosuvastatin on carotid intima-media thickness in children with heterozygous familial hypercholesterolemia: the CHARON study (hypercholesterolemia in children and adolescents taking rosuvastatin open label). Circulation.

[6] Buonuomo PS, Macchiaiolo M, Leone G, Valente P, Mastrogiorgio G, Gnazzo M (2018). Treatment of homozygous familial hypercholesterolaemia in paediatric patients: a monocentric experience. Eur J Prev Cardiol.

[7] FOR EPOIG, CHILDREN RRI (2011). Expert panel on integrated guidelines for cardiovascular health and risk reduction in children and adolescents: summary report. Pediatrics.

[8] Pediatrics AAo (1992). American Academy of Pediatrics National Cholesterol Education Program: report of the expert panel on blood cholesterol levels in children and adolescents. Pediatrics.

[9] de Ferranti SD, Steinberger J, Ameduri R, Baker A, Gooding H, Kelly AS (2019). Cardiovascular risk reduction in high-risk pediatric patients: a scientific statement from the American Heart Association. Circulation.

[10] Hovsepian S, Kelishadi R, Djalalinia S, Farzadfar F, Naderimagham S, Qorbani M (2015). Prevalence of dyslipidemia in Iranian children and adolescents: a systematic review. J Res Med Sci.

[11] Kant AK, Graubard BI (2012). Race-ethnic, family income, and education differentials in nutritional and lipid biomarkers in US children and adolescents: NHANES 2003–2006. Am J Clin Nutr.

[12] Freedman DS, Bowman BA, Otvos JD, Srinivasan SR, Berenson GS (2002). Differences in the relation of obesity to serum triacylglycerol and VLDL subclass concentrations between black and white children: the Bogalusa heart study. Am J Clin Nutr.

[13] Bentley AR, Rotimi CN (2017). Interethnic differences in serum lipids and implications for cardiometabolic disease risk in African ancestry populations. Glob Heart.

[14] Kelishadi R, Motlagh ME, Bahreynian M, Gharavi MJ, Kabir K, Ardalan G, et al. Methodology and early findings of the assessment of determinants of weight disorders among Iranian children and adolescents: the childhood and adolescence surveillance and prevention of adult noncommunicable disease-IV study. Int J Prev Med. 2015;6. 10.4103/2008-7802.162953 PMC4564901 26425332

[15] Peterson AL, McBride PE. A review of guidelines for dyslipidemia in children and adolescents. WMJ. 2012;20(8). 23362704

[16] Phan BAP, Toth PP (2014). Dyslipidemia in women: etiology and management. Int J Women’s Health.

[17] Ekta G, Tulika MG (2016). Risk factor distribution for cardiovascular diseases among high school boys and girls of urban Dibrugarh, Assam. J Family Med Prim Care.

[18] He H, Pan L, Du J, Liu F, Jin Y, Ma J, et al. Prevalence of, and biochemical and anthropometric risk factors for, dyslipidemia in children and adolescents aged 7 to 18 years in China: A cross-sectional study. Am J Hum Biol. 2019:e23286. 10.1002/ajhb.23286 31254309

[19] Giuliano ICB, MSSdA C, SFTd F, MMdS P, Zunino JN, RQdC R (2005). Serum lipids in school kids and adolescents from Florianópolis, SC, Brazil: healthy Floripa 2040 study. Arq Bras Cardiol.

[20] Shim YS, Baek JW, Kang MJ, Oh YJ, Yang S, II TH. Reference values for the triglyceride to high-density lipoprotein cholesterol ratio and non-high-density lipoprotein cholesterol in Korean children and adolescents: the Korean National Health and nutrition examination surveys 2007-2013. J Atheroscler Thromb. 2016:35634. 10.5551/jat.35634 PMC5221496 27373984

[21] Nielsen TRH, Lausten-Thomsen U, Fonvig CE, Bøjsøe C, Pedersen L, Bratholm PS (2017). Dyslipidemia and reference values for fasting plasma lipid concentrations in Danish/north-European white children and adolescents. BMC Pediatr.

[22] Eissa MA, Mihalopoulos NL, Holubkov R, Dai S, Labarthe DR (2016). Changes in fasting lipids during puberty. J Pediatr.

[23] Slhessarenko N, Jacob CM, Azevedo RS, Fontes CJ, Novak GV, Andriolo A (2015). Serum lipids in Brazilian children and adolescents: determining their reference intervals. BMC Public Health.

